# Using Bifactor Twin Modeling to Assess the Genetic and Environmental Dimensionality of Adult ADHD Symptoms

**DOI:** 10.1007/s10519-024-10204-y

**Published:** 2024-10-30

**Authors:** Jacob Knyspel, Geneviève Morneau-Vaillancourt, Thalia C. Eley

**Affiliations:** 1 Social, Genetic & Developmental Psychiatry Centre, Memory Lane, London, SE5 8AF UK; 2 Institute of Psychiatry, Psychology & Neuroscience, London, UK; 3 King’s College London, London, UK

**Keywords:** ADHD, Twin study, Adults, Heritability, Behavioral genetics, Bifactor

## Abstract

Attention Deficit Hyperactivity Disorder (ADHD) is a common and heritable neurodevelopmental condition that has been the subject of a wealth of genetics research. Because ADHD has an early age of onset, most of this work has focused on children, meaning that less is known about the genetics of ADHD in adults. Additionally, while much research has assessed the heritability of ADHD as a general dimension, less has assessed the heritability of individual subtypes (inattention, hyperactivity) or symptoms of ADHD. It therefore remains unclear whether the genetic factors underlying ADHD symptoms conform to a unidimensional or multidimensional structure. The aim of this study was to assess the genetic and environmental dimensionality of adult ADHD symptoms. We analyzed data from 10,454 twins of the Twins Early Development Study, who provided self-reports of ADHD symptoms using the Conners scale at age 21 years. The data conformed well to a psychometric bifactor model, providing support for a general dimension of ADHD in addition to secondary dimensions for inattention and hyperactivity. However, a bifactor independent pathway twin model provided support for a general dimension only at the level of non-shared environmental effects and not additive genetic or shared environmental effects. This suggests that symptoms of ADHD cluster together under a general dimension of non-shared environmental effects, although the two subtypes of ADHD (inattention and hyperactivity) are meaningfully genetically distinct. We found the overall heritability of ADHD to be 40%, comparable with previous estimates for adult ADHD symptoms. Our results provide useful insights into the genetic and environmental architecture of specific ADHD symptoms.

**Supplementary Information:**

The online version contains supplementary material available at 10.1007/s10519-024-10204-y.

Attention Deficit Hyperactivity Disorder (ADHD) is a common and heterogeneous neurodevelopmental condition characterized by attentional problems, hyperactivity, and impulsivity (American Psychiatric Association 2013). It has a mean age of diagnosis of approximately 7–9 years (Rocco et al. 2021), although it is believed to be noticeable to caregivers as early as 2 years of age (van Lieshout et al. 2016), making it one of the earliest onset psychiatric conditions. ADHD is especially prevalent in young people, with global prevalence estimates of 7.6% in children aged 3–12 years and 5.6% in adolescents aged 12–18 years (Salari et al. 2023). Unlike other neurodevelopmental conditions, such as autism spectrum disorder and intellectual disability, the symptomology of ADHD often changes considerably with age. Many young people with ADHD no longer meet diagnostic thresholds later in life (Vos and Hartman 2022), with the prevalence of ADHD decreasing to 2.5% in adults (Song et al. 2021). This might reflect a true decrease in ADHD symptoms with age, although making such a conclusion is limited by the fact that studies have often relied on retrospective reporting during adulthood (e.g. Vos and Hartman 2022). Research has also suggested that symptoms of ADHD can emerge for the first time during adulthood (Agnew-Blais et al. 2016; Moffitt et al. 2015), despite childhood symptoms typically being required for an ADHD diagnosis. This has led researchers to consider whether childhood and adulthood ADHD are qualitatively distinct from one another (Castellanos 2015). However, the role that additional factors such as lifestyle changes or assessment methods (diagnosis vs. symptoms) might also explain observed differences between childhood and adulthood ADHD requires further investigation, ideally via prospective longitudinal studies. Despite the wealth of available pharmacological and psychological interventions for ADHD in both children and adults (Caye et al. 2019), the prognosis for ADHD remains poor in many cases. Treatment adherence is notably low among people with ADHD (Biederman et al. 2019), and even among those who adhere to treatment, ADHD remains associated with adverse life outcomes including unemployment, substance abuse, and criminality (Erskine et al. 2016). ADHD is thus a highly concerning condition in need of further research regarding its etiology, pathophysiology, and treatment (Cattoi et al. 2022).

In children, twin and family studies have shown that genetic factors account for a substantial proportion of the variance in ADHD, with consistent heritability estimates of 70–80% (Faraone and Larsson 2019). In adolescents and adults, who have been the focus of fewer studies, heritability estimates are less consistent. Most adolescent and adult twin studies have used self-report ADHD symptom scales, producing heritability estimates of only 30–40% (e.g., Boomsma et al. 2010; Haberstick et al. 2008; Larsson et al. 2013; Li et al. 2020; van den Berg et al. 2006). These findings have led researchers to conclude that a qualitative distinction exists between childhood and adulthood ADHD, which would be congruent with the changing symptomology of ADHD with age. A further possibility is that the heritability of ADHD simply decreases with age and that environmental factors account for a larger proportion of the variance in ADHD over time (Boomsma et al. 2010). However, there is also evidence suggesting that the heritability of clinically diagnosed ADHD in young adulthood, assessed via public health records, is around 70% (Larsson et al. 2014), a much greater heritability estimate than those from studies relying on self-reports (30–40%). These inconsistent findings may be partly attributable to how ADHD is assessed across studies, such as through self-reports, parent-reports, and health records (see Brikell et al. 2015; Faraone and Larsson 2019). Indeed, when combining self-reports and parent-reports into latent factors, Chang et al. (2013) found that the heritability of attention problems remained consistent from ages 8 to 20. Regardless, this difference highlights the need for research focusing on the genetics of ADHD specifically in adults. Better understanding of how the influence of genetics changes with age might help to explain why the presentation of ADHD often differs among children and adults. It might also help to inform interventions for the condition that are better tailored to child and adult populations, improving support for people with ADHD throughout their lifespan.

If researchers wish to use twin data to study the genetics of ADHD symptoms, several statistical models are relevant. The simplest is a univariate model which estimates genetic and environmental effects on a total ADHD symptom score. This univariate model has by far been the most common method for estimating the heritability of adult ADHD symptoms (e.g. Boomsma et al. 2010; Larsson et al. 2013; van den Berg et al. 2006). An alternative is the common pathway model (Kendler et al. 1987), which estimates genetic and environmental effects on a latent factor which itself loads onto numerous observed variables such as ADHD symptoms. Constructing a latent factor out of observed variables can help to minimize measurement error among these variables, potentially providing a more precise and less biased estimate of heritability. A further alternative is the independent pathway model (Kendler et al. 1987). In this model, shared genetic and environmental effects among variables are estimated directly on each variable, rather than via a latent factor. Unlike the common pathway model, the independent pathway model does not assume that the shared genetic and environmental effects on a set of variables are mediated through the same latent factor. It is therefore useful for assessing whether the genetic and environmental effects on variables have unique factor structures (see Franić et al. 2013).

If multiple sets of variables are included in common and independent pathway models, such as symptoms reflecting the inattention and hyperactivity subtypes of ADHD, useful extensions to these models become possible. One extension that has seen little use in research is the inclusion of a general latent factor that represents the unidimensionality of all the observed variables in a model (e.g., Pelt et al. 2022; Zakharin & Bates, 2022; see Fig. 1). Structural equation models which include general latent factors, called “bifactor” models (Holzinger and Swineford 1937), have become increasingly popular in research as they provide an ideal method for modeling the covariances of variables at multiple levels of generality (Reise et al. 2023). This includes covariances at the general level (what a variable shares with all other variables) and group level (what a variable shares with a subset of other variables). They are especially relevant for ADHD, since much psychometric research has established that ADHD symptoms conform well to bifactor models (for a review, see Arias et al. 2018). This means that while ADHD symptoms can load onto latent factors representing different subtypes of the condition, they typically load most strongly onto a shared general factor. It indicates that even after accounting for the presence of subtypes, ADHD symptoms remain unidimensional enough that a general dimension of ADHD is psychometrically meaningful. This general dimension could be said to correspond to a combined-type ADHD presentation that is meaningfully distinct from subtype-specific presentations (Milich et al. 2001). If general latent factors are included in twin models, these models can be used to provide insight into the genetic and environmental dimensionality of symptoms. These terms refer to the extent to which symptoms of a condition share genetic and environmental effects within specific subsets (e.g., inattention, hyperactivity), across all of subsets together (e.g., overall ADHD), or both.

**Fig. 1:**
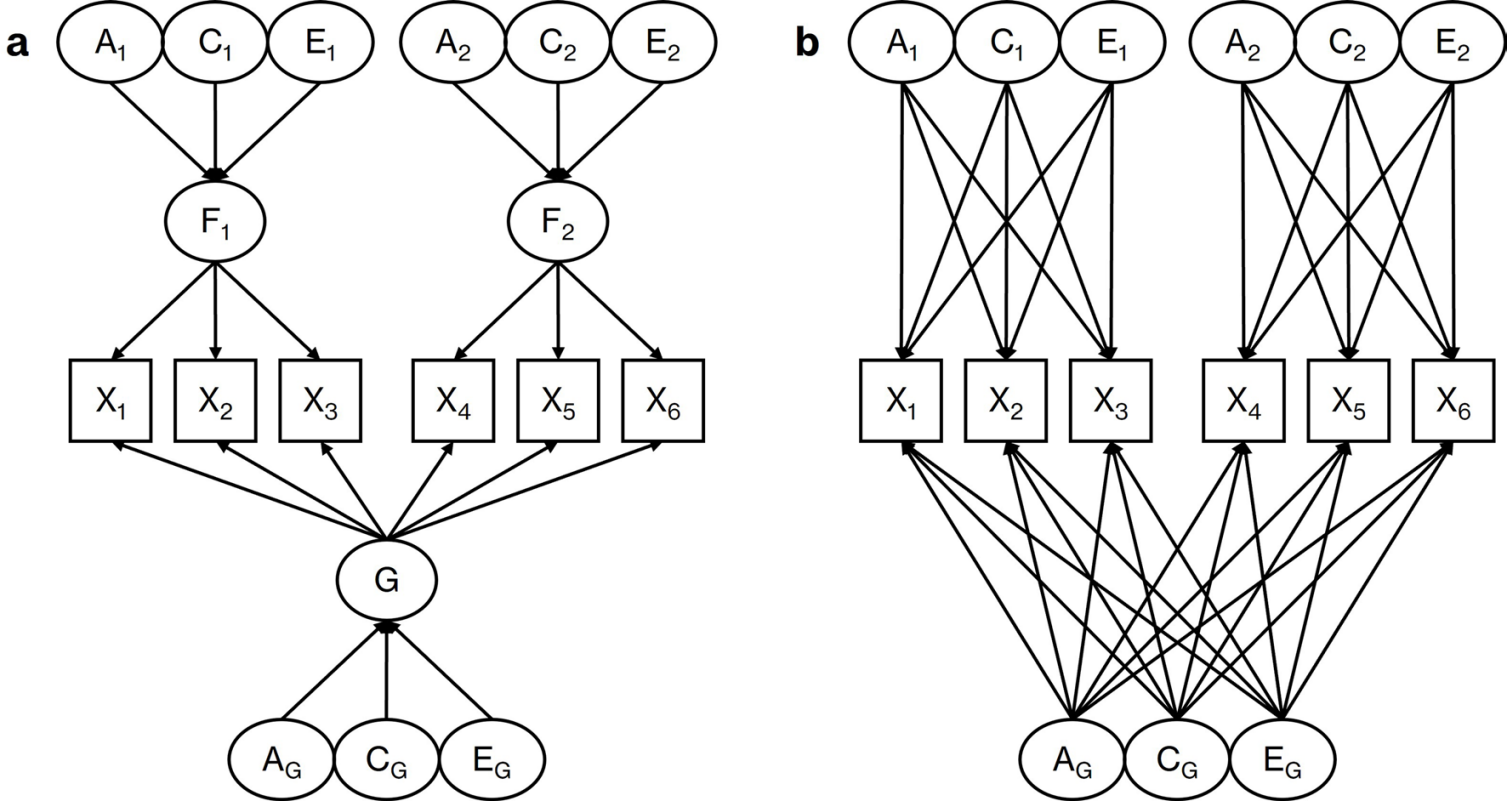
Bifactor common pathway model (**a**) and bifactor independent pathway model (**b**), both of which include general latent factors. *Note* X = Observed variable; A = Additive genetic effect; C = Shared environmental effect; E = Non-shared environmental effect; F = Latent factor; G = General latent factor

Our aim with this study was to assess the genetic and environmental dimensionality of ADHD symptoms in a sample of young adult twins as measured using the Conners self-report symptom scale (Conners 2008). First, we performed a psychometric analysis in which an overall bifactor structure was fitted to the ADHD symptoms. Based on previous findings (Arias et al. 2018), we hypothesized that the data would conform to this bifactor structure well. Second, we performed genetic analyses in which a bifactor common pathway model and bifactor independent pathway model were used to the assess the genetic and environmental dimensionality of ADHD symptoms in young adulthood. We had no specific hypothesis as to whether the genetic and environmental dimensionality of ADHD symptoms would be equivalent, as no studies have yet examined the genetic and environmental dimensionality of ADHD symptoms using bifactor twin models. Based on existing literature (e.g., McLoughlin et al. 2007; Nikolas and Burt 2010), we hypothesized that the overall heritability of the two ADHD subtypes, and by extension an ADHD general factor, would be roughly equivalent.

## Method

### Sample

Existing data were analyzed from the Twins Early Development Study (TEDS; Lockhart et al. 2023), an ongoing twin cohort study investigating the development of 13,759 pairs of twins born in the years 1994–1996 in England and Wales. Specifically, self-reported ADHD symptom data collected at age 21 were used. This included data from 10,454 twins, 35.1% of whom were monozygotic and 64.9% of whom were dizygotic. The mean age of the sample was 22.85 (SD = 0.88). Of the sample, 59.9% and 40.1% were assigned female and male at birth respectively. TEDS has ethical approval from Kings College London Research Ethics Committee (References: PNM/09/10–104 and HR/DP-20/21–22060). Prior to accessing the data, this study was pre-registered at https://osf.io/emx8y.

### Measures

ADHD symptoms were measured using the short self-report version of the Conners scale (3rd edition; Conners 2008). This scale includes 20 items assessing ADHD symptoms (e.g. “It is hard for me to sit still”) which are self-rated on an ordinal scale from 0 (“Not true at all”) to 3 (“Definitely true”) in response to the following question: “To what extent do the following statements accurately describe you?”. This question was not timeframe specific. There are two subscales: an 11-item subscale for inattention symptoms and a 9-item subscale for hyperactivity symptoms. In this sample, the internal consistency of the Conners scale was found to be strong (Cronbach’s α = 0.89, McDonald’s ω = 0.89), as was the internal reliability of the inattention subscale (α = 0.88, ω = 0.88) and hyperactivity subscale (α = 0.82, ω = 0.83).

### Psychometric Analysis

The psychometric analysis assessed how well the Conners scale conforms to a bifactor structure comprising one general latent factor (overall ADHD) and two specific latent factors (inattention and hyperactivity). Direct Schmid-Leiman orthogonalisation (Waller 2018) was used to estimate this bifactor structure, using the *R* package “fungible” (Waller et al. 2023). This method was chosen because it performed best in a recent large simulation study (Giordano and Waller 2020). To minimize bias, only the Conners scale item scores from one randomly selected twin per pair were included. Among these twins, 17% had missing data, resulting in a sample of 4338 twins following listwise deletion. The meaningfulness of the general latent factor within the structure was evaluated in terms of standardized factor loadings (λ) in addition to the internal consistency (ω_h_, cutoff = > 0.5; McDonald 1999), construct reliability (*H*-index, cutoff = > 0.7; Hancock & Muller, 2001), and explained common variance (ECV; Ten Berge and Sočan 2004) of the general latent factor (for a review of these bifactor-specific indices, see Rodriguez et al. 2016). These indices were selected because model fit statistics do not necessarily provide useful information regarding whether a general latent factor has a meaningful interpretation within a bifactor model (Bonifay and Cai 2017; Reise et al. 2016). Based on general guidelines (e.g., Pituch and Stevens 2016), standardized factor loadings greater than 0.4 were considered to be adequate. Since the bifactor structure was orthogonal in nature, meaning that factors were constrained to be uncorrelated, correlations between factors were not assessed.

### Genetic Analyses

We then examined the genetic and environmental dimensionality of the Conners scale items by conducting and comparing bifactor common pathway and independent pathway models. The models included additive genetic, shared environmental, and non-shared environmental effects (i.e., ACE models; see Rijsdijk and Sham 2002; for a description of the twin method). We coded models using the *R* packages “OpenMx” (Boker et al. 2011) and “umx” (Bates et al. 2019). They were run using the SLSQP optimizer, available in the “OpenMx” package, and 95% confidence intervals were bootstrapped with 1000 replications for all statistics of interest. As with the psychometric analysis, 17% of twins had missing data, which were handled via full information maximum likelihood estimation. The age, sex, and cohort of twins were included as covariates by residualizing them out of the data prior to the fitting of the twin models. As with the psychometric analysis, we assessed the meaningfulness of the general latent factors within each twin model (i.e., the overall general latent factor within the bifactor common pathway model and the separate genetic and environmental general latent factors within the bifactor independent pathway model) in terms of standardized factor loadings, internal consistency, construct reliability, and explained common variance. For each twin model, we tested alternative versions in which additive genetic and/or shared environmental effects were dropped to assess the redundancy of these effects and identify the most parsimonious models. We compared models using five fit statistics: the Root Mean Squared Error of Approximation (RMSEA), Comparative Fit Index (CFI), -2 Log-Likelihood (-2LL), Akaike Information Criterion (AIC), and Bayesian Information Criterion (BIC). Based on previous research (Hu and Bentler 1999), we used cutoffs of RMSEA < 0.06 and CFI > 0.95 to indicate strong model fit. We also performed likelihood ratio tests to compare the different versions of each twin model.

### Data Availability

The data used in this study are not openly available. TEDS data can be accessed by researchers by application, see: https://www.teds.ac.uk/researchers/teds-data-access-policy.

## Results

### Psychometric Analysis

Among the entire sample, the mean Conners scale total score was 13.5 (SD = 8.8) out of a possible 60. The estimated psychometric bifactor structure showed that the general latent factor had adequate internal consistency (ω_h_ = 0.62, cutoff = > 0.5) and construct reliability (*H*-index = 0.88, cutoff = > 0.7), explaining 52% of the common variance across items. The standardized factor loadings within the bifactor structure are presented in Table 1. All items except for two (“Dislikes complex tasks”, “Prefers being on the go”) had adequate loadings (λ > 0.4) onto the general factor, with most items (60%) loading more strongly onto the general factor than subscale factors. All items correctly loaded onto their intended subscale factor more strongly than the other subscale factor. Together these findings indicate that the Conners scale item data conformed sufficiently well to a bifactor structure to be suitable for subsequent genetic analyses with bifactor twin models.

**Table 1:** Standardized factor loadings (λ) of Conners scale items within the psychometric bifactor structure

Item	Brief Item Description	Subscale	General	Inattention	Hyperactivity
1	Struggles paying attention	Inattention	0.53	0.50	0.04
2	Makes mistakes	Inattention	0.40	0.38	0.01
3	Struggles focusing on tasks	Inattention	0.59	0.57	0.02
4	Struggles focusing on conversations	Inattention	0.56	0.50	0.06
5	Struggles following instructions	Inattention	0.56	0.52	0.04
6	Struggles finishing tasks	Inattention	0.52	0.55	-0.03
7	Struggles with organisation	Inattention	0.46	0.53	-0.07
8	Dislikes complex tasks	Inattention	0.34	0.38	-0.04
9	Loses things	Inattention	0.47	0.38	0.09
10	Gets distracted	Inattention	0.59	0.46	0.13
11	Forgets things	Inattention	0.50	0.47	0.03
12	Struggles sitting still	Hyperactivity	0.53	0.14	0.39
13	Leaves seat inappropriately	Hyperactivity	0.57	0.19	0.38
14	Feels restless	Hyperactivity	0.56	0.19	0.36
15	Struggles remaining quiet	Hyperactivity	0.58	0.10	0.48
16	Prefers being on the go	Hyperactivity	0.29	-0.08	0.37
17	Talks too much	Hyperactivity	0.41	-0.13	0.54
18	Blurts out answers to questions	Hyperactivity	0.47	-0.09	0.56
19	Struggles waiting turns	Hyperactivity	0.53	-0.03	0.55
20	Interrupts others	Hyperactivity	0.45	-0.02	0.48

*Note* Brief item descriptions are not the full item statements

### Genetic Analyses

Model fit statistics for each twin model, as well as the results of the likelihood-ratio tests used to compare nested models, are presented in Table 2. As this table shows, bifactor independent pathway models had consistently better fit statistics than bifactor common pathway models, which indicates that the shared genetic and environmental effects on Conners scale items were best modeled as independent and not mediated by a common latent factor. The best fitting bifactor common pathway model was the AE version, while the best fitting bifactor independent pathway model was the ACE version. Only the best fitting bifactor independent pathway model met the pre-specified cutoffs for strong model fit (RMSEA < 0.06, CFI > 0.95).

**Table 2:** Model fit statistics and likelihood ratio tests for the bifactor common pathway and bifactor independent pathway twin models

Model	RMSEA	CFI	-2LL	AIC	BIC	LRT *p*
Bifactor common pathway model						
ACE	0.027	0.906	336795.3	337060.0	337488.6	
**AE**	**0.027**	**0.907**	**336795.3**	**337011.8**	**337365.0**	**> 0.999**
CE	0.030	0.888	338045.8	338262.3	338615.4	< 0.001
E	0.029	0.888	338045.8	338214.5	338491.8	< 0.001
Bifactor independent pathway model						
**ACE**	**0.018**	**0.961**	**333107.8**	**333523.9**	**334182.6**	
AE	0.020	0.952	333773.3	334061.1	334525.6	< 0.001
CE	0.023	0.933	335014.4	335302.3	335766.8	< 0.001
E	0.029	0.888	338045.8	338208.4	338475.7	< 0.001

*Note* A = Additive genetic effects; C = Shared environmental effects; E = Non-shared environmental effects; RMSEA = Root mean squared error of approximation; AIC = Akaike information criterion; BIC = Bayesian information criterion; -2LL = -2 Log-Likelihood; LRT = Likelihood-ratio test. Likelihood-ratio tests compared models to their corresponding ACE versions. **Bold** indicates the best fitting version

The genetic and environmental effect estimates from the models are presented in Table 3. As this table shows, the bifactor common pathway model produced heritability estimates of 40% for the ADHD general factor, 42% for the inattention subscale factor, and 33% for the hyperactive subscale factor. Although the heritability of inattention items appeared larger than the heritability of hyperactive items, this difference was not statistically significant (see Table 3 for confidence intervals). In the bifactor independent pathway model, the general factor accounted for 50% of the heritability of the Conners scale items on average, while the inattention and hyperactivity factors both accounted for 19%. The remaining 12% was accounted for by genetic effects that were unique to individual items. In contrast to the common pathway model, the general factor in the independent pathway model therefore accounted for a larger proportion of the overall heritability of Conners scale items than both the inattention and hyperactivity factors combined.

**Table 3:** Genetic and environmental effect estimates from best fitting bifactor common pathway and bifactor independent pathway twin models

Model	A [95% CI]	C [95% CI]	E [95% CI]
Bifactor common pathway model			
*General*	0.40 [0.36, 0.45]	-	0.60 [0.55, 0.64]
*Inattention*	0.42 [0.36, 0.47]	-	0.58 [0.53, 0.64]
*Hyperactivity*	0.33 [0.26, 0.39]	-	0.67 [0.61, 0.74]
Bifactor independent pathway model			
*General*	0.08 [0.05, 0.12]	0.02 [0.00, 0.04]	0.18 [0.15, 0.22]
*Inattention*	0.03 [0.02, 0.04]	0.01 [0.00, 0.01]	0.10 [0.09, 0.11]
*Hyperactivity*	0.03 [0.02, 0.04]	0.01 [0.01, 0.01]	0.05 [0.04, 0.05]
*Unique*	0.02 [0.02, 0.03]	0.00 [0.00, 0.01]	0.46 [0.46, 0.47]

*Note* A = Additive genetic effect; E = Non-shared environmental effect; CI = Confidence interval (bootstrapped with 1000 iterations). For the bifactor common pathway model, the genetic and environmental effect estimates for each latent factor (general, inattention, hyperactivity) are reported. For the bifactor independent pathway model, genetic and environmental effects are broken down into the effects that were either shared among factors (general, inattention, hyperactivity) or unique to items. These effects were calculated as the means of the squared standardized effects across items. The squared standardized effects for each individual item are reported in Supplementary Table 1

The indices used to evaluate the meaningfulness of the general latent factors within the bifactor independent pathway model are reported in Table 4, along with those from the psychometric analysis as a reference. Only the non-shared environmental general factor was found to have adequate indices (ω_h_ = 0.67, *H*-index = 0.86), these indices being similar to those from the psychometric analysis. The additive genetic general factor had a marginally adequate ω_h_ value but inadequate *H*-index (ω_h_ = 0.51, *H*-index = 0.68), and the shared environmental general factor had wholly inadequate indices (ω_h_ < 0.01, *H*-index = 0.16). This indicates that the psychometric bifactor structure of Conners scale items was sufficiently reflected only in their non-shared environmental effects. By extension, it suggests that the concept of a general dimension for either additive genetic or shared environmental effects was not supported by the data. The standardized factor loadings within each bifactor structure from the twin models are reported in Supplementary Tables 2–5.

**Table 4:** General factor indices for the ACE bifactor independent pathway twin model, in addition to those from the prior psychometric analysis

Model	ω_h_	H-Index	ECV
Bifactor independent pathway model			
*Additive genetic effects*	0.51	0.68	0.59
*Shared environmental effects*	< 0.01	0.16	0.31
*Non-shared environmental effects*	0.67	0.86	0.59
Psychometric analysis (reference)			
*Psychometric bifactor structure*	0.62	0.88	0.52

*Note* ω_h_ = Omega hierarchical (cutoff = > 0.5); *H*-index = Construct reliability (cutoff = > 0.7); ECV = Explained common variance

## Discussion

The aim of this study was to assess the genetic and environmental dimensionality of adult ADHD symptoms, or the extent to which ADHD symptoms share genetic and environmental effects across subtypes as opposed to within subtypes. We applied bifactor twin models to ADHD symptom data measured using the Conners scale from adult twins aged 21–26. First, we showed that the data conformed well to a psychometric bifactor structure with three latent factors (general, inattention, hyperactivity), in line with previous findings about ADHD symptoms (Arias et al. 2018). This suggests that at a phenotypic level, a general dimension of ADHD that can be measured beyond the individual domains of inattention and hyperactivity is highly replicable. This general dimension is theoretically important because it provides a useful reference point for understanding why some people with ADHD exhibit both symptoms of inattention and hyperactivity (i.e., a combined-type ADHD presentation) while others do not.

We then used a bifactor common pathway model and a bifactor independent pathway model. The bifactor independent pathway model fit the data better than the bifactor common pathway model, suggesting that genetic and environmental effects on adult ADHD symptoms were best modeled as independent and not mediated by a common latent factor. Within the bifactor independent pathway model, only non-shared environmental effects, and not additive genetic or shared environmental effects, conformed well to a bifactor structure. This is because the general latent factors estimated for additive genetic and shared environmental effects did not reach the cutoffs required to be considered meaningful. It suggests that the non-shared environmental effects on ADHD symptoms are unidimensional enough that a general dimension of ADHD (i.e., a combined-type presentation) be considered meaningful, even after accounting for subtypes, although the same is not true of genetic and shared environmental effects. This has important implications for understanding the etiology of ADHD. Unidimensionality at the level of non-shared environmental effects indicates that environmental risk factors that can differ among twins, such as childhood head trauma (Adeyemo et al. 2014) or eczema (van der Schans et al. 2017), have a roughly equivalent association with inattention and hyperactivity, thereby contributing to a combined-type presentation. Conversely, a lack of unidimensionality at the level of shared environmental effects indicates that environmental risk factors shared by twins, such as maternal pre-pregnancy obesity (Jenabi et al. 2019), might be more uniquely associated with either inattention or hyperactivity (for a review of environmental risk factors for ADHD, see Kim et al. 2020). In this way, the use of twin modeling revealed important differences between certain types of environmental risk factors (shared vs. non-shared) in their relationship to ADHD symptoms.

As for genetics, the findings from the bifactor independent pathway model suggest that ADHD symptoms in young adulthood are genetically multidimensional enough that a general dimension of ADHD is not as meaningful genetically as phenotypically. In other words, the findings suggest that the two subtypes of ADHD can be considered meaningfully genetically distinct. Findings from the broader ADHD literature support this conclusion. For example, although the genetic correlation between ADHD subtypes is large (Kuntsi et al. 2014; McLoughlin et al. 2007), the genetic correlations among other psychiatric phenotypes, such as between depression and anxiety, are often larger (e.g., Morneau-Vaillancourt et al. 2020). Similarly, some estimates of the genetic correlation between ADHD subtypes (e.g., McLoughlin et al. 2007) have been roughly equivalent to estimates of the genetic correlations between ADHD and other neurodevelopmental conditions (Andersson et al. 2020). If the subtypes of ADHD are genetically multidimensional and have a genetic correlation that is equivalent to or lower than the genetic correlations among other psychiatric phenotypes, this has important implications. Genetic separation between symptoms of inattention and hyperactivity implies the existence of distinct biological pathways that could be targeted by more symptom-specific medications or behavioral interventions. Future research might therefore aim to explore how genetic differences between inattention and hyperactivity can be translated to improved treatments. A more extreme implication might be that inattention and hyperactivity should be considered distinct conditions altogether (see Grizenko et al., 2009). If the subtypes of ADHD truly reflected a single condition, it might be expected that ADHD symptoms would be both genetically and environmentally unidimensional, which was not observed. Considering the results of this and previous studies, findings that the genetic overlap between inattention and hyperactivity is lower than might be expected have now been made in both children (Kuntsi et al. 2014; McLoughlin et al. 2007) and young adults. This suggests that the genetic overlap between inattention and hyperactivity might be one aspect of the genetics of ADHD that remains fairly consistent with age.

The overall heritability of self-reported adult ADHD symptoms was estimated to be 40% in this study. This estimate is consistent with those from previous adult twin studies of self-reported ADHD symptoms (Faraone and Larsson 2019). As previously mentioned, it is notably lower than the estimated heritability of ADHD in children (Faraone and Larsson 2019). This study therefore provided additional evidence that heritability might be an aspect of the genetics of ADHD that changes with age, unlike the genetic overlap between inattention and hyperactivity. However, it is again important to note that this change in heritability might instead reflect methodological issues such as a distinction between diagnoses and self-report scales. Through the use of a bifactor common pathway model, this study operationalized ADHD symptomology as a latent variable, rather than an observed total symptom score. The use of latent variables allows statistical models to better account for the measurement error which frequently occurs among self-report items (Palmer et al. 2002). Since measurement error can inflate estimates of non-shared environmental effects in twin models (van den Berg et al. 2007), accounting for measurement error had the potential to increase the estimated heritability of self-reported adult ADHD symptoms. This would have made the heritability estimate closer to that which has been observed for clinically diagnosed adult ADHD (Larsson et al. 2014). Given that the heritability estimate from this study is consistent with those from previous studies, it is likely that measurement error has had a minimal impact on previous estimates of the heritability of self-reported adult ADHD symptoms.

There are a several limitations to this study. First, twin data are sometimes considered ungeneralizable to the broader population. Several researchers have argued that social and cultural factors might cause families with twins to be systematically different to other families (Mönkediek et al. 2020; Schwabe et al. 2017). Evidence for the existence of systematic differences between twins and non-twins specifically in the presentation of ADHD is mixed (Gjone et al., 1995; Levy et al. 1996), and so the severity of this limitation is unclear. It is therefore important to explore these issues using other genetically informative designs. Second, our analyses assumed no gene-environment interplay in the etiology of ADHD. A wealth of research has found that gene-environment interplay influences the emergence and persistence of ADHD symptoms (Nigg et al. 2010). Importantly, gene-environment interplay can bias the results of twin models (Purcell 2002; Verhulst and Hatemi 2013), including the bifactor twin models used in this study. Future research might therefore aim to combine bifactor twin models with components that allow for the modeling of gene-environment interplay, such as environmental moderators (Purcell 2002). Third, we used only self-reported measures of ADHD symptoms. Future research might aim to expand upon these analyses to include additional raters (e.g. family members) to ensure clarity over presentation of symptoms across different environments (e.g. home, school, workplace).

Despite these limitations, this study provides new insight into the genetic and environmental dimensionality of adult ADHD symptoms. Importantly, we found that although adult ADHD symptoms have substantial psychometric unidimensionality, they have limited genetic unidimensionality. One possible conclusion from this study is that adult ADHD symptoms are genetically multidimensional enough that the subtypes of ADHD be considered meaningfully genetically distinct. In genome-wide association studies, power might be improved by undertaking analyses at the subtype level rather than at the level of ADHD diagnosis per se. ADHD is just one example among many psychiatric conditions for which the use of bifactor twin models might be useful to assess genetic and environmental unidimensionality. Symptom scales used to measure many psychiatric conditions are known to be fitted well by bifactor models, including psychotic disorders (Anderson et al. 2018) and mood disorders (Simms et al. 2008). Bifactor twin models might also provide insight into genetic unidimensionality across conditions, especially those for which their phenotypic unidimensionality has previously been assessed using bifactor modeling, such as ADHD and oppositional defiant disorder (Burns et al. 2020; Lee et al. 2016). This would in turn provide invaluable insight into the contribution of genetics to the risk of common ADHD comorbidities. In this way, the use of bifactor twin models is a desirable method for assessing the dimensionality of conditions such as ADHD in greater detail.

## Electronic Supplementary Material

Below is the link to the electronic supplementary material.

Supplementary Material 1

Supplementary Material 2

## Data Availability

The data used in this study are not openly available. Twins Early Development Study data can be accessed by researchers by application, see: https://www.teds.ac.uk/researchers/teds-data-access-policy.
