# Supplementary material for: Using Bifactor Twin Modeling to Assess the Genetic and Environmental Dimensionality of Adult ADHD Symptoms
Source: Behav Genet. 2024 Oct 30;55(1):1–11. doi: 10.1007/s10519-024-10204-y (PMC11790749; doi:10.1007/s10519-024-10204-y)
Supplement: Supplementary file 2 — Supplementary Material 2 [file 10519_2024_10204_MOESM2_ESM.docx]

## Supplementary Figure 1

Bifactor common pathway model fitted to Conners scale data.


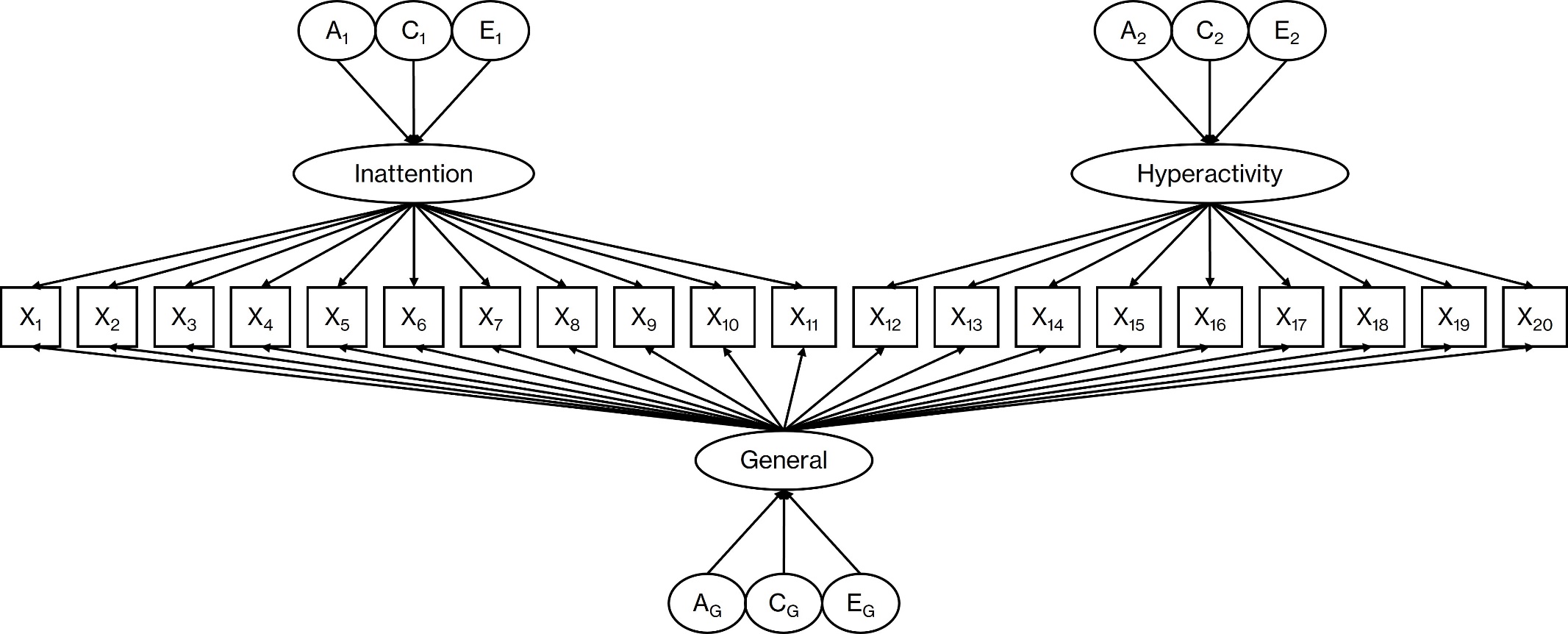


*Note*. X = Conners scale item; A = Additive genetic effect; C = Shared environmental effect; E = Non-shared environmental effect; G = General latent factor.

## Supplementary Figure 2

Bifactor independent pathway model fitted to Conners scale data.


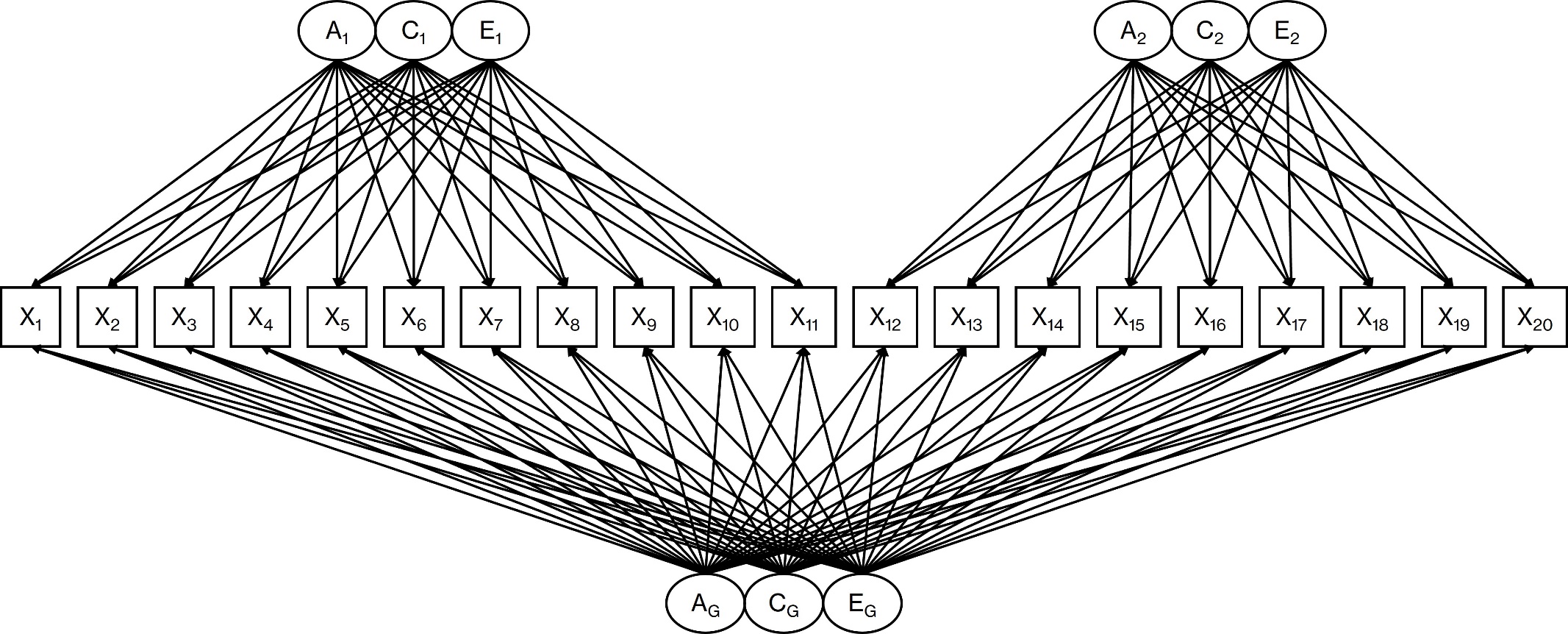


*Note*. X = Conners scale item; A = Additive genetic effect; C = Shared environmental effect; E = Non-shared environmental effect; G = General latent factor.
